# Mechanism of oxidative stress p38MAPK-SGK1 signaling axis in experimental autoimmune encephalomyelitis (EAE)

**DOI:** 10.18632/oncotarget.17057

**Published:** 2017-04-12

**Authors:** Liang Wang, Bin Li, Mo-Yuan Quan, Lin Li, Yuan Chen, Guo-Jun Tan, Jing Zhang, Xiao-Peng Liu, Li Guo

**Affiliations:** ^1^ Department of Neurology, The Second Hospital of Hebei Medical University, Shijiazhuang, Hebei 050000, China; ^2^ Key Laboratory of Hebei Neurology, Shijiazhuang, Hebei 050000, China; ^3^ Department of Neurology, Tongren Hospital of Capital Medical University, Beijing, Hebei 100088, China; ^4^ Department of Pediatrics, The Second Hospital of Hebei Medical University, Shijiazhuang, Hebei 050000, China; ^5^ Department of Neurosurgery, The Second Hospital of Hebei Medical University, Shijiazhuang, Hebei 050000, China

**Keywords:** oxidative stress, p38MAPK-SGK1, EAE, multiple sclerosis

## Abstract

**Background:**

Multiple sclerosis (MS), a complex disease associated with multifocal demyelination of the central nervous system and poorly understood etiology. It has been previously indicated that many factors, including oxidative stress and p38MAPK-SGK1 pathway, contribute to the pathogenesis of MS.

**Methods:**

This study, using an experimental autoimmune encephalomyelitis (EAE) model system, was aimed at investigating the molecular mechanisms determining interaction p38MAPK-SGK1 pathway and oxidative stress in MS pathogenesis. C57BL/6 mice was immunized with MOG35-55 peptide for EAE induction, which was followed by determination of the effect of treatment with classic p38 inhibitor SB203580 and antioxidant tempol on the development and progression of EAE.

**Results:**

Our experiments showed a dynamic change of immune inflammation, oxidative stress and p38MAPK-SGK1 pathway involvement in EAE demonstrating that p38MAPK-SGK1 pathway and oxidative stress contribute to the demyelination in central nerve system caused by Th17 inflammatory responses in a synergistic way. The administration of SB203580 and Tempol both markedly suppressed the progression of EAE. Furthermore, tempol showed a strong inhibiting effect to the p38MAPK-SGK1 pathway similar to SB203580 suggesting that oxidative stress exacerbates EAE via the activation of p38MAPK-SGK1 pathway.

**Conclusion:**

Cumulatively, our results show that oxidative stress p38MAPK-SGK1 signaling pathway may be a central player in EAE and even in MS.

## INTRODUCTION

Multiple sclerosis (MS), which affects one million people worldwide, is a central nervous system (CNS) associated autoimmune disease [1]. The etiology of MS is not completely understood [1]. It is characterized by demyelination, axonal damage, progressive impaired neurological function, and an inflammatory infiltrate [1]. The classical *in vivo* model to study MS is the experimental autoimmune encephalomyelitis (EAE) model. EAE exhibits all properties of MS, inclusive of demyelination, axonal damage, and neurological dysfunction, as well as sensitivity to clinically approved therapies against MS [1, 2]. Even though the precise etiology of MS is not yet known, inflammation induced by antigen-specific T cell immunity involving both Th1 and Th17 cells seems to be a central driver of MS pathogenesis [1, 3–5]. The inflammatory cascade is initiated by the CD4+ T cells in the CNS microenvironment, and subsequent secretion of toxicants like reactive oxygen species (ROS) by other immune and resident cells mediate the killing of oligodendrocytes. Neurological dysfunction in MS results as a result of axonal (oligodendrocyte-myelin-axon) damage [6, 7]. Hence, it is not surprising that oxidative stress driven by inflammation is also a critical node in both EAE and MS pathogenesis [1, 8].

High levels of reactive oxygen species (ROS) is the major cause of oxidative stress, which can disrupt the blood-brain barrier (BBB). This leads to a cascade of events – leukocyte migration that in turn mediates myelin phagocytosis, and resultant axonal injury [1, 9]. Antioxidants based therapeutic interventions thus show promising results in attenuating development and alleviating progression of MS [10]. Moreover, many other factors such as Vitamin D deficiency and infectious mononucleosis have been proved to increase the risk of MS [11, 12].

Although a variety of factors may indeed be involved in MS, the current data does not elucidate the central regulator(s). The efficacy of either immune-modulating agents or anti-oxidants therapy is limited [8, 13]. However, how exactly neuroinflammation and oxidative stress cooperatively contribute to MS pathogenesis is largely unknown. It is still unclear which pathway is mediated by oxidative stress to achieve pathogenesis and progress in MS and EAE. It is known that stress activates the p38 MAP kinase (MAPK)-SGK1 signaling pathway that cumulatively generate the inflammatory responses [1]. Activivation of p38 MAPK has been shown to be required for production of IL-17 production by Th17 cells, both *in vitro* and *in vivo*, mandatorily requires p38 MAPK activation [14, 15]. Importantly, a number of recent studies have identified MAPK activation as a central player in MS and EAE [16]. We infer p38MAPK-SGK1 may be the converging point of such pathogenic mechanisms. Oxidative stress contributes to the pathogenesis of MS and EAE via p38MAPK-SGK1 activation of central nervous system. Oxidative stress-p38MAPK-SGK1 axis may regulate key immunopathogenic mechanisms underlying EAE.

In this study, we immunized C57BL/6 mice with MOG35-55 peptide to induce EAE and determined the effect of treatment with classic p38 inhibitor SB203580 and antioxidant tempol on the development and progression of EAE. Moreover, we determined the potential mechanisms underlying the action of them in inhibiting the development and progression of EAE in mice.

## RESULTS

### Treatment with either p38 inhibitor or antioxidant reduces the severity of EAE in mice

**Figure 1:**
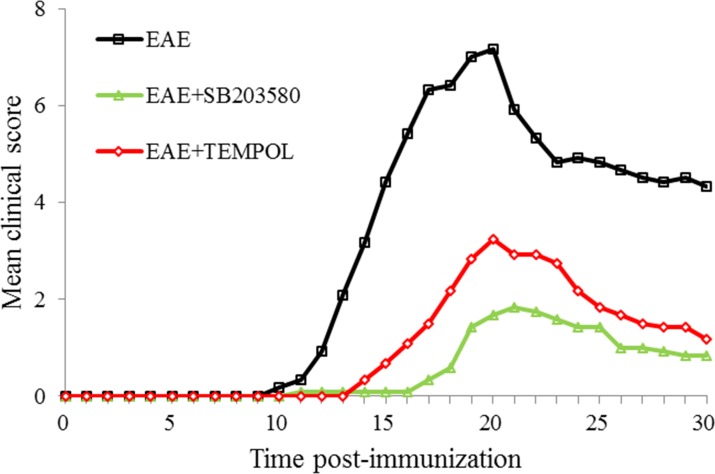
Each of the two treatments inhibits the development and progression of EAE in mice C57BL/6 mice were immunized with MOG35-55 in CFA to induce EAE and the mice were randomly treated with SB203580, tempol, or vehicle alone. The development and severity of EAE in individual mice were monitored longitudinally. Data are expressed as the mean ± SD of the clinical scores of each group of mice. n=12 per experimental group.

C57BL/6 mice were immunized with MOG35-55 in CFA to induce EAE before being randomly treated with SB203580, tempol, or vehicle alone. The development and severity of clinical signs in the different groups of mice were monitored longitudinally in Figure 1. The control EAE group of mice began to show clinical signs on day 10 (mean onset time after immunization: 12.58 days) post immunization and all of them developed EAE eventually. The clinical signs of mice from EAE group got fastigium on day 20 post immunization. All the animals entered remission period before day 30 post immunization (Supplementary Tables 1 and 2). The mean score of EAE group was 2.08 on day 13 and 7.17 on day 18 while 4.33 on day 30.

The mice from intervention groups were treated by SB203580 or tempol once daily after immunization. The rate of EAE development in both SB203580-treated mice and tempol-treated mice was significantly lower than that in the EAE group (P<0.05) while the onset time and peak time were delayed (P<0.05). More importantly, the mean clinical scores in these two intervention groups were significantly lower than that in the EAE mice (P<0.05).

### Treatment with p38 inhibitor and antioxidant both mitigate inflammatory infiltrates and demyelination in the CNS

**Figure 2:**
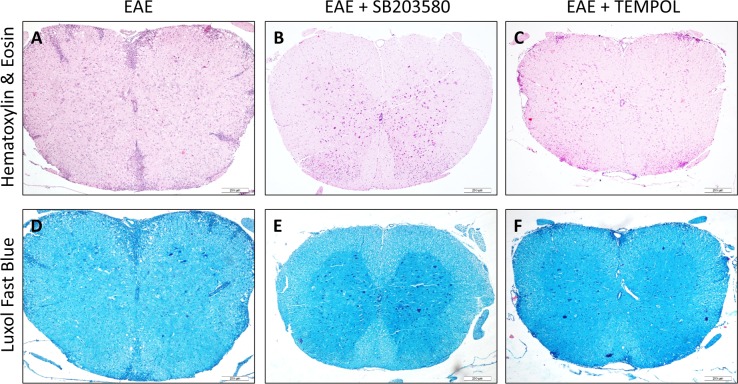
Each of the two treatments inhibits the development and progression of EAE in mice **(A-C)** Comparison of HE staining in different groups. There were obvious reduced numbers of inflammatory infiltrates in the spinal cords of the intervention groups of mice. **(D-F)** Comparison of LFB staining in different groups. The degree of demyelination in the intervention groups of mice was significantly lower than that in the EAE mice. Data shown are representative images from each group or expressed as the mean ± SD of each group (n = 6). A,D: EAE, B,E: SB203580, C,F: TEMPOL. Scale bar = 200 μm.

We examined the pathologic changes in the spinal cords from the EAE groups on day 13, 20, and 30 post-immunization by histology compared with the healthy control mice. Inflammatory infiltrate was not detected in the CNS and exhibited regularly arranged myelin in the spinal cord in the healthy control mice. In comparison, both inflammatory infiltrates and demyelination was prominent in the spinal cords of the vehicle-treated EAE mice. The inflammatory infiltrates were aggravated along with the change of EAE clinical signs and the mean degree of inflammation on day 20 was higher than that on either day 13 or day 30 (Supplementary Figure 1). Compared with the vehicle-treated EAE mice, there were obvious reduced numbers of inflammatory infiltrates in the spinal cords of the SB203580-treated mice and tempol-treated mice. On day 20, the inflammation score of SB203580 group was lower than that of tempol group (P<0.05) (Figure 2A–2C, Supplementary Table 3).

The mean degree of demyelination also got worse with the course of disease from onset to remission period (Supplementary Figure 2). Semi-quantitative analysis revealed that the pathological scores of demyelination in the SB203580-treated mice and tempol-treated mice were significantly lower than that in the EAE mice (Figure 2D-2F, Supplementary Table 4).

**Figure 3:**
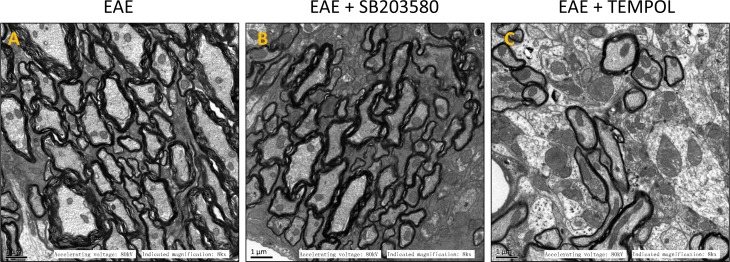
Images of electron microscope for different groups **(A)** EAE, **(B)** EAE + SB203580, **(C)** EAE + TEMPOL.

The myelin sheath of lumber intumescentia became loose and irregularly on day 13 in the group of vehicle-treated EAE mice. In fastigium, the structure of white matter got unclear and some sections displayed signs of broken, disaggregation or even deficiency of myelin. The axon became atrophied and degenerate at the same time (Supplementary Figure 3). On the sections from EAE group, the myelin sheath became much looser, more axons showed atrophy and degeneration, and the boundary between grey matter and white matter become unclear on day 30 (Figure 3A). There were not obvious change for the myelin sheath and axon in the sections of treatment groups on day 13. The damage of myelin and axons were mitigated during peak and remission period due to the p38MAPK inhibitor and antioxidant (Figure 3B, 3C). Taken together, the results exhibited weakened inflammatory infiltration, reduced demyelinating area and lightened axonal injury in central nerve system of mice from intervention groups.

### Treatment with p38 inhibitor and antioxidant both modulate inflammatory responses

**Figure 4:**
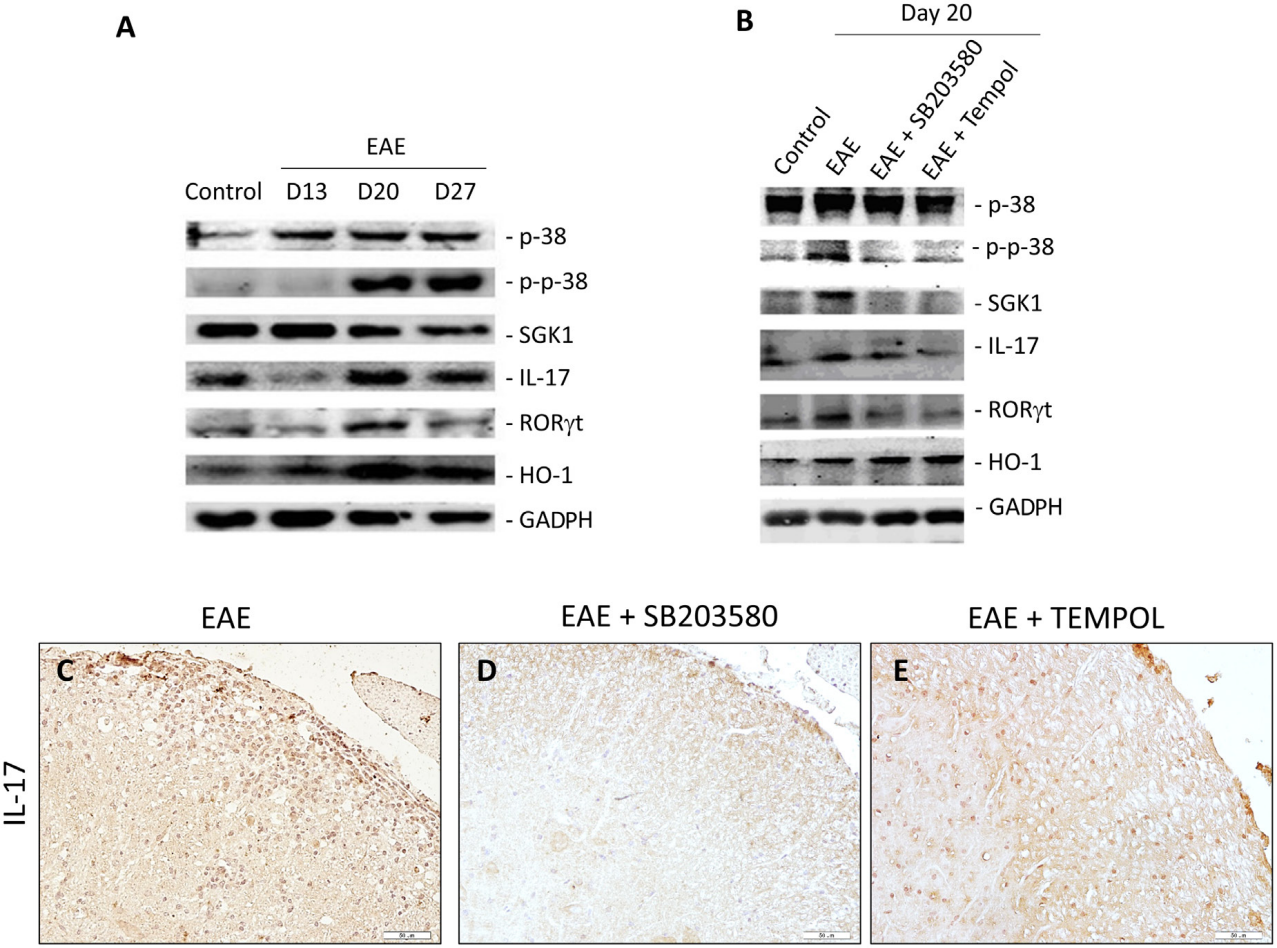
**(A, B)** Representative photographs of Western blot analysis of p38, P-p38, SGK, IL-17, RORγt, and HO-1 in the lumbar enlargement of EAE mice at different time points **(A)**, and different intervention groups **(B)**. **(C, D, E)** Immunohistochemical detection of IL-17 in lumber intumescentia of spinal cords of mice from different groups. The expression of IL-17 in spinal cords increased in intervention groups.

We explored the variation of T cell immunity in control vehicle treated EAE mice by immunohistochemistry and Western blot assays. The transcription factor RORγt is an important regulator of Th17 differentiation. While there were low levels of RORγt and IL-17 expression in the spinal cord at day 13, much higher levels of RORγt and IL-17 expression were observed at day 20 in the EAE group of mice. The levels of expression were decreased at 30 days post immunization (Figure 4A, Supplementary Table 5).

Then we examined the impact of treatment with SB203580 and tempol on Th17 cell immunity in EAE mice on day 20. We observed that both of the reagents effectively mitigated EAE-related increase in the levels of RORγt and IL-17 expression in spinal cords, related to that in the EAE mice (Figure 4B, 4C–4E, Supplementary Table 6).

### Treatment with p38 inhibitor and antioxidant both inhibit the p38MAPK-SGK1 pathway

The p38MAPK-SGK1 signaling pathway has been identified as a central player in MS and EAE. We examined the variation of p38MAPK-SGK1 pathway in EAE mice by Western blot assays. The expression of p-p38MAPK and SGK1 in spinal cords of EAE mice increased from onset time to the peak time (Figure 4A). The levels of SGK1 expression decreased at 30 days post immunization (Figure 4A). There was no change in the content of p38MAPK during this time. We next explored the expression levels of p-p38MAPK and SGK1 in spinal cords on day 20 to examine the impact of SB203580 and tempol. The levels of p-p38MAPK and SGK1 decreased significantly in the intervention groups of mice compared with that in vehicle-treated EAE mice (Figure 4B). There was no obvious difference in the levels of p38MAPK between the EAE and treatment groups. Taken together, treatment with p38 inhibitor and antioxidant both inhibit the p38MAPK-SGK1 pathway.

### Both p38MAPK inhibitor and antioxidant can suppress oxidative stress and up-regulate HO-1

**Figure 5:**
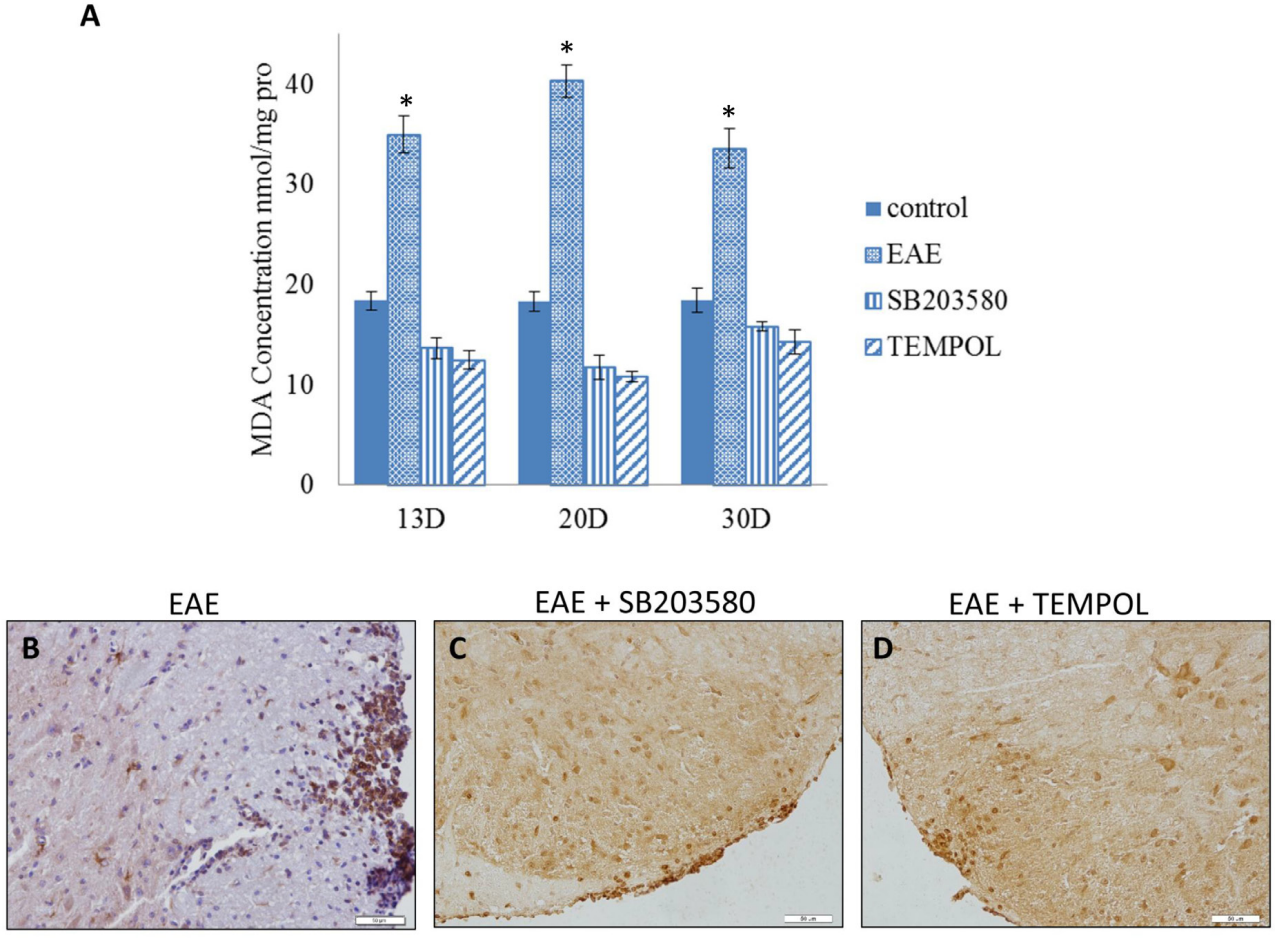
**(A)** The content of MDA in the spines of mice of different groups on day 13, day 20 and day 30 (n = 3 per group). *compared with other groups, P<0.05. **(B, C, D)** Immunohistochemical detection of HO-1 in lumber intumescentia of spinal cords of mouse from different groups.

We measured the MDA expression to detect the level of oxidative stress during the course. We next explored the expression of HO-1 in spinal cords of EAE mice by western blot assays at different time points. The mean MDA concentration began to increase after the onset and got to a high level at the clinical peak stage of EAE while decreased in remission. The level of HO-1 continued to rise along the EAE course and got to the top on day30 (Figure 4A, Supplementary Table 7). The level of MDA was significantly lower in either SB203580 or tempol treated group than that in EAE group at all the time points (Figure 5A). Besides, treatment with both SB203580 and tempol increased the expression of HO-1 on day 20 (Figure 5B-5D). Collectively, all these interventions lead to up-regulated HO-1 contributing to the antioxidant activity in mice.

## DISCUSSION

Experimental autoimmune encephalomyelitis (EAE) is well documented as robust model to study MS pathogenesis [17]. CD4 T-helper (Th) lymphocytes are central in regulating host immune responses as well as inflammatory and autoimmune diseases [18]. Aberrant homeostasis in the pro-inflammatory T cell responses is a critical driver of MS pathogenesis, both in humans and EAE in rodents [3, 19–21]. T helper 17 (Th17) cells produce IL-17, IL-21, and IL-22, each one critical in mounting an immune response. For the same reasons, Th17 lymphocytes can also confer susceptibility to autoimmune diseases [4, 5]. Indeed, both Th1 and Th17 cells have been shown to be critical in MS pathogenesis [22, 23]. RORγt is selectively expressed in Th17 cells and is effective in specifying the Th17 phenotype [24].

ROS is an important regulator of a multitude of cellular processes. However, impaired ROS homeostasis results in oxidative stress, in turn leading to damage to proteins, lipids and nucleic acids [25]. ROS-mediated oxidative stress contribute to MS by acting on distinct pathological processes including BBB disruption, which in turn enhances leukocyte migration and subsequent myelin phagocytosis, oligodendroglial damage and axonal injury [9, 26].

To counteract the detrimental effects of ROS the central nervous system is endowed with a powerful antioxidant defense mechanism to protect cells against ROS mediated toxicity and to maintain tissue redox balance. This stress response includes enhanced protein expression of Nrf2/ARE-regulated antioxidant enzymes like superoxide dismutases (SODs), peroxiredoxins (Prxs) and heme oxygenases (HOs) [27, 28]. Previous studies show that HO-1 or downstream products of heme metabolism may be interesting targets for MS treatment [29, 30]. Enhanced expression of endogenous antioxidant enzymes like HO-1 is suggestive of ongoing oxidative stress and activation of antioxidant defense mechanism in MS and EAE lesions and functions as a protective mechanism against ROS-mediated cellular toxicity [31]. A well-recognized indicator of peroxidation in the CNS is MDA [32].

Although the exact source of ROS may be ambiguous, the importance of ROS in the activation of p38 is not. Antioxidants can completely abrogate its activation [33]. The p38MAP kinase (MAPK) signaling pathway is essential for *in vitro* and *in vivo* IL-17 production by regulating IL-17 synthesis in CD4 T cells [34, 35]. Serum glucocorticoid kinase 1 (SGK1), a serine/ threonine kinase is one of the substrates of p38MAPK. SGK1 is under transcriptional control of numerous stimuli [16]. Oxidative stress stimulates Sgk expression through a p38/MAPK-dependent pathway [36]. Evidence from mouse and human studies supports the role of p38MAPK-SGK1 in regulating key immunopathogenic mechanisms underlying autoimmune inflammatory disease of the central nervous system [37].

Our research showed a dynamic change of immune inflammation, oxidative stress and p38MAPK-SGK1 pathway in EAE. Along with the worsening of EAE clinical signs, the induction of Th17 cells was boosted, oxidative stress leveled up while expression of p38MAPK and SGK1 increased. Either p38MAPK-SGK1 pathway or oxidative stress showed consistency for EAE course, suggesting a strong correlation between them. Our data demonstrates that p38MAPK-SGK1 pathway and oxidative stress may contribute to the demyelination in central nerve system caused by Th17 inflammatory responses in a synergistic way and indicates the need for additional investigations to exploit the interaction between them.

Pathogenic T cell responses against myelin antigens expressed in the CNS lead to MS. In the central nervous systems (CNS), activation of the p38MAPK pathway constitutes a key step in the development of several diseases including MS [38]. P38MAPK contributes to the differentiation of pathogenic T cells initiating the inflammatory cascade and is crucially involved in the tissue destruction and pathology mediated by other immune and resident cells. In two papers published on Nature, the authors link the process of Th17 differentiation with the protein kinase enzymes p38MAPK and SGK1 [38, 39].

Although oxidant stress may indeed worsen autoimmune disease, the data provided do not establish exactly which pathways oxidant stress works on to achieve this [40]. Our data has demonstrated a strong correlation between oxidant stress and p38MAPK-SGK1 pathway in EAE. Taken together, we infer that oxidative stress may contributes to the pathogenesis of EAE via p38MAPK-SGK1 activation of central nervous system. Oxidative stress-p38MAPK-SGK1 axis may regulate key immunopathogenic mechanisms underlying EAE. We evaluated whether treatment with the p38MAPK inhibitor or multifunctional antioxidant tempol affects the ensuing EAE. SB203580 is a prototypical ATP competitive imidazole based inhibitor and has been identified as a cytokine-suppressive anti-inflammatory drug [38]. Tempol is a superoxide dismutase mimetic. Tempol, by changing the redox status of the inflammatory microenvironment within the CNS, ameliorate murine viral encephalomyelitis [41]. However, the effects of tempol on MS are not known.

We discover that the administration of SB203580 or Tempol markedly suppressed the progression of EAE. The mean clinical scores in the mice from treatment groups were lower than that in the vehicle-treated control EAE mice throughout the observation period. Histological examination showed weakened inflammatory infiltration, reduced demyelinating areas and lightened axonal injury in central nerve system. Results from immunohistochemistry and western blot analysis suggest the Th17 immunoreaction was much weakened correlating with diminished activation of p38MAPK-SGK1 and oxidative stress. We show in this study that the activation of p38MAPK is critical for the development of EAE, since inhibition of the p38MAPK pathway with selective small-molecule inhibitors prevented the development of clinical disease when administered prophylactically. Furthermore, ROS were involved in the p38MAPK-controled T cell inflammation and ROS scavenging attenuated this process. Taken together, we suggest that oxidative stress exacerbates EAE via the activation of p38MAPK-SGK1 pathway particularly given that antioxidant therapy also inhibited the p38MAPK-SGK1 pathway strongly in our research. P38MAPK-SGK1 pathway acts as the converging point of oxidative stress and Th17 inflammatory responses in central nerve system. We propose the following mechanism underlying the oxidant stress and the pathogenesis of EAE: oxidant stress activates the p38MAPK-SGK1 pathway, which reinforces the Th17 phenotype and thus induces the pathological injury and neurological dysfunction. Oxidative stress p38MAPK-SGK1 pathway may be a central player in EAE and even MS.

To conclude, the observation of this study suggests a strong correlation between oxidant stress and p38MAPK-SGK1 pathway in T cell mediated autoimmune inflammation. Our data showed oxidative stress contributes to the pathogenesis of EAE via p38MAPK-SGK1 activation of central nervous system. Oxidative stress p38MAPK-SGK1 axis regulates key immunopathogenic mechanisms underlying EAE, an area that will warrant additional investigation in the future.

## MATERIALS AND METHODS

### Animals

Female C57BL/6 mice of 8 - 10 weeks of age were obtained from Vital River (Beijing, China) and kept in a specific pathogen-free facility with cycles of 12 hours dark/light and free access to food and water [1]. The experimental protocols were approved by the Institutional Animal Care and Use Committee of Hebei Medical University.

### Induction and assessment of EAE and treatment

EAE was induced as described previously [1]. Briefly, mice were injected subcutaneously with 250 μg MOG35-55 peptide (Lysine Bio-system, Xian, China) emulsified in complete Freund's adjuvant (CFA, Sigma, St. Louis, USA) containing 4 mg/ml of heat-killed *Mycobacterium tuberculosis* (Difco Laboratories, Detroit, MI, USA). At 0 hour and 48 hours after immunization, mice were injected intraperitoneally with 500 ng pertussis toxin (Alexis, San Diego, CA, USA).

Randomized treated intraperitoneally once per day with vehicle or drugs - SB203580 at 5 mg/kg body weight, TEMPOL at 25 mg/kg body weight. The mice were examined daily for clinical signs of EAE and scored as follows: the scale ranged from 0 to 15 and is the sum of the state of the tail and all of the four limbs. For the tail the following scoring was followed - 0 reflected no signs, 1 reflected a half paralyzed tail, and score of 2 reflected fully paralyzed tail. For each of the hind-or forelimbs, each assessed separately, 0 signified no signs, score 1 signified weak or altered gait, score 2 signified paresis, while a score of 3 signified fully paralyzed limb. A fully paralyzed quadriplegic animal would thus a score of 14. Mortality equals a score of 15.

### Histology and immunohistochemistry

Perfused through the left cardiac ventricle with saline, plus 0.5 M EDTA was performed on mice from each group for 5–10 minutes, subseuentlyfollowed by fixation with cold 4% paraformaldehyde (PFA) in 0.1 M phosphate buffer (pH 7.4). Thereafter spinal cords were dissected out and post-fixed in 4% PFA for 3–4 hours and processed for paraffin embedding. Five μm paraffin CNS sections of control, EAE, SB203580, and Tempol EAE treated mice were used for histological quantification of neurological damage in EAE mice. Two different stains were used to detect inflammatory infiltrates and demyelination. Sections were stained with Luxol Fast Blue (LFB) for the evaluation of demyelination [1]. The degrees of inflammation and demyelination on three non-serial sections of each mouse were assessed semi-quantitatively in a blinded manner [1, 39].

IHC was used to assess the expression of p38, p-p38, SGK1, IL-17, RORγt and HO-1 expression in the spinal cord tissue as described before [1]. The sections were incubated with anti-p38 MAPK, anti-p-p38 MAPK, anti-SGK1 (Cell Signaling Technologies, USA), anti-IL-17, anti-RORγt, or anti-HO-1 (Bioworld, USA) at 4°C overnight.

### Electron microscopy

Electron microscopy was performed as described before [1].

### Measurement of malondialdehyde (MDA)

The degrees of oxidative stress in the spines were assessed for the contents of malondialdehyde (MDA) as has been described before [1]. The concentrations of MDA were represented as nmol/mg proteins.

### Western blot analysis

Western blot analysis was done as described before [1]. The relative levels of target protein to control expression were quantified by densitometric scanning.

### Statistical analysis

Kruskal- Wallis test was used to analyze the difference in clinical scores among the different experimental groups, whereas the Mann-Whitney U test was used to assess the difference in clinical scores between two therapy groups of mice. The onset rates of EAE between groups were analyzed by chi-square test. All other statistical comparisons among groups were examined using Student's t test or ANOVA analysis followed by SNK-q test. Unless otherwise indicated, data were presented as mean ± standard deviation (SD). *P* values less than 0.05 were considered statistically significant.

## SUPPLEMENTARY FIGURES AND TABLES



## Abbreviations

MS: Multiple sclerosis
EAE: experimental autoimmune encephalomyelitis
CNS: central nervous system
ROS: reactive oxygen species
BBB: blood–brain barrier
HE: hematoxylin & eosin
LFB: Luxol Fast Blue
PFA: paraformaldehyde
SD: standard deviation
Th: T-helper
ODs: dismutases
Prxs: peroxiredoxins
HOs: heme oxygenases
MDA: Malondialdehyde
SGK1: Serum glucocorticoid kinase 1
