# Supplementary material for: Mechanism of oxidative stress p38MAPK-SGK1 signaling axis in experimental autoimmune encephalomyelitis (EAE)
Source: Oncotarget. 2017 Apr 12;8(26):42808–16. doi: 10.18632/oncotarget.17057 (PMC5522107; doi:10.18632/oncotarget.17057)
Supplement: Supplementary file 1 [file oncotarget-08-42808-s001.pdf]

Mechanism of oxidative stress p38MAPK-SGK1 signaling axis in experimental autoimmune encephalomyelitis (EAE)

SUPPLEMENTARY FIGURES AND TABLES

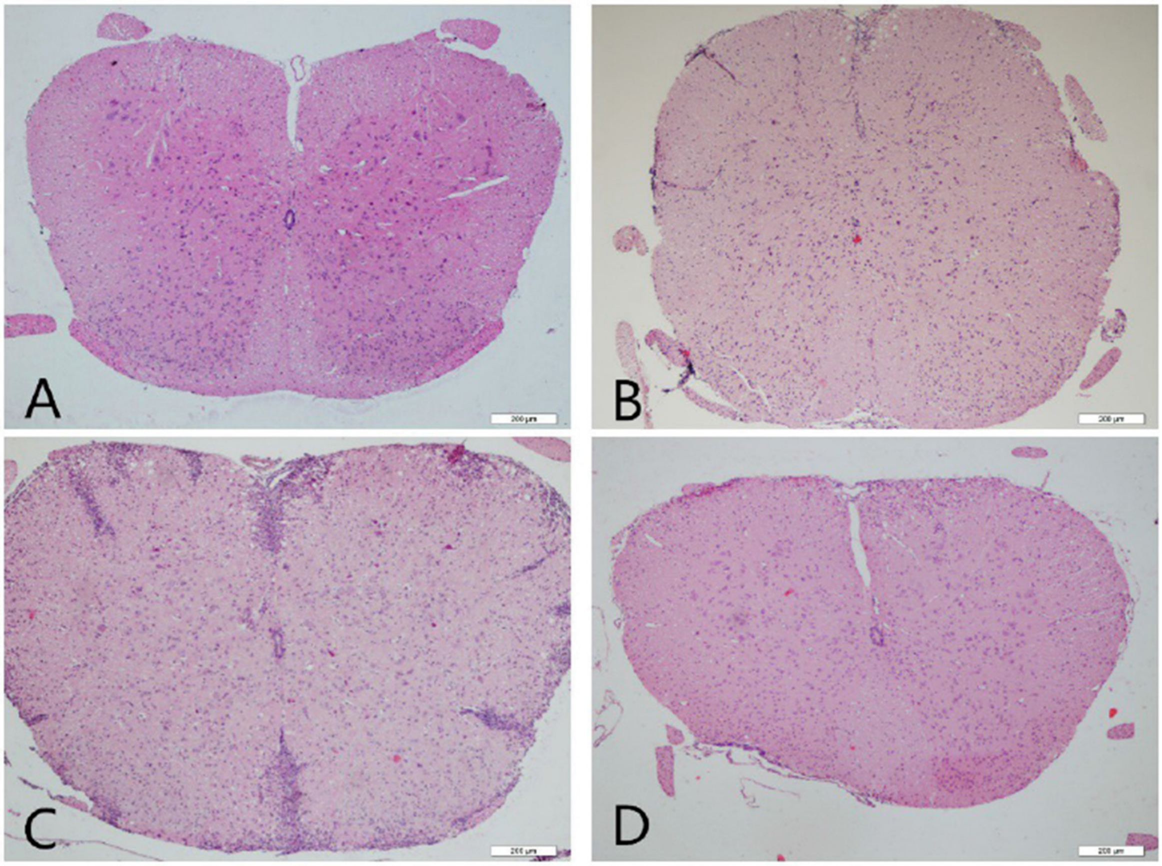

Supplementary Figure 1: Comparison of HE staining at different time points. The inflammatory infiltrates were aggravated along with the change of EAE clinical signs. Data shown are representative images from each group or expressed as the mean ± SD of each group (n = 6). **(A)** control, **(B)** EAE 13d, **(C)** EAE 20d, **(D)** EAE 30d. Scale bar = 200 µm.

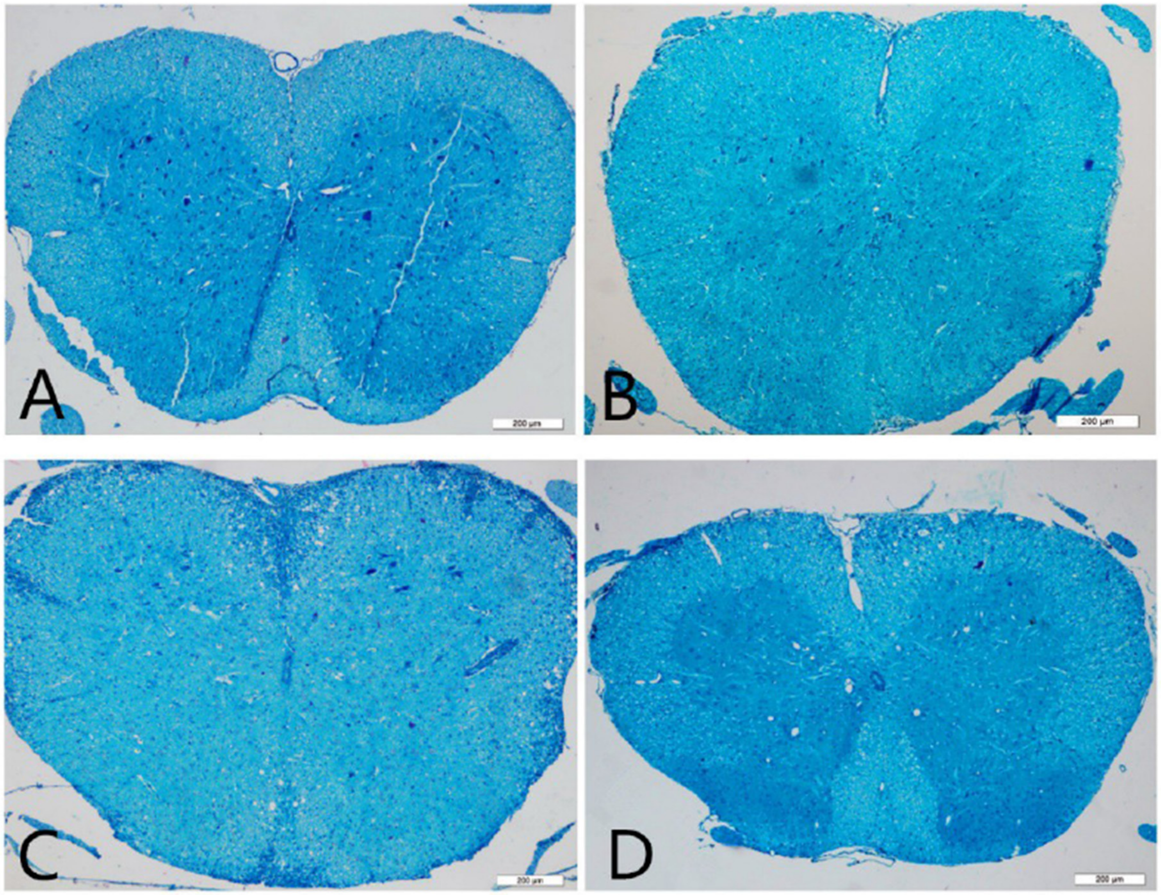

Supplementary Figure 2: Comparison of LFB staining different time points. The mean degree of demyelination got worse with the course of disease from onset to remission period. Data shown are representative images from each group or expressed as the mean ± SEM of each group (n = 6). **(A)** control, **(B)** EAE 13d, **(C)** EAE 18d, **(D)** EAE 30d. Scale bar = 200 μm.

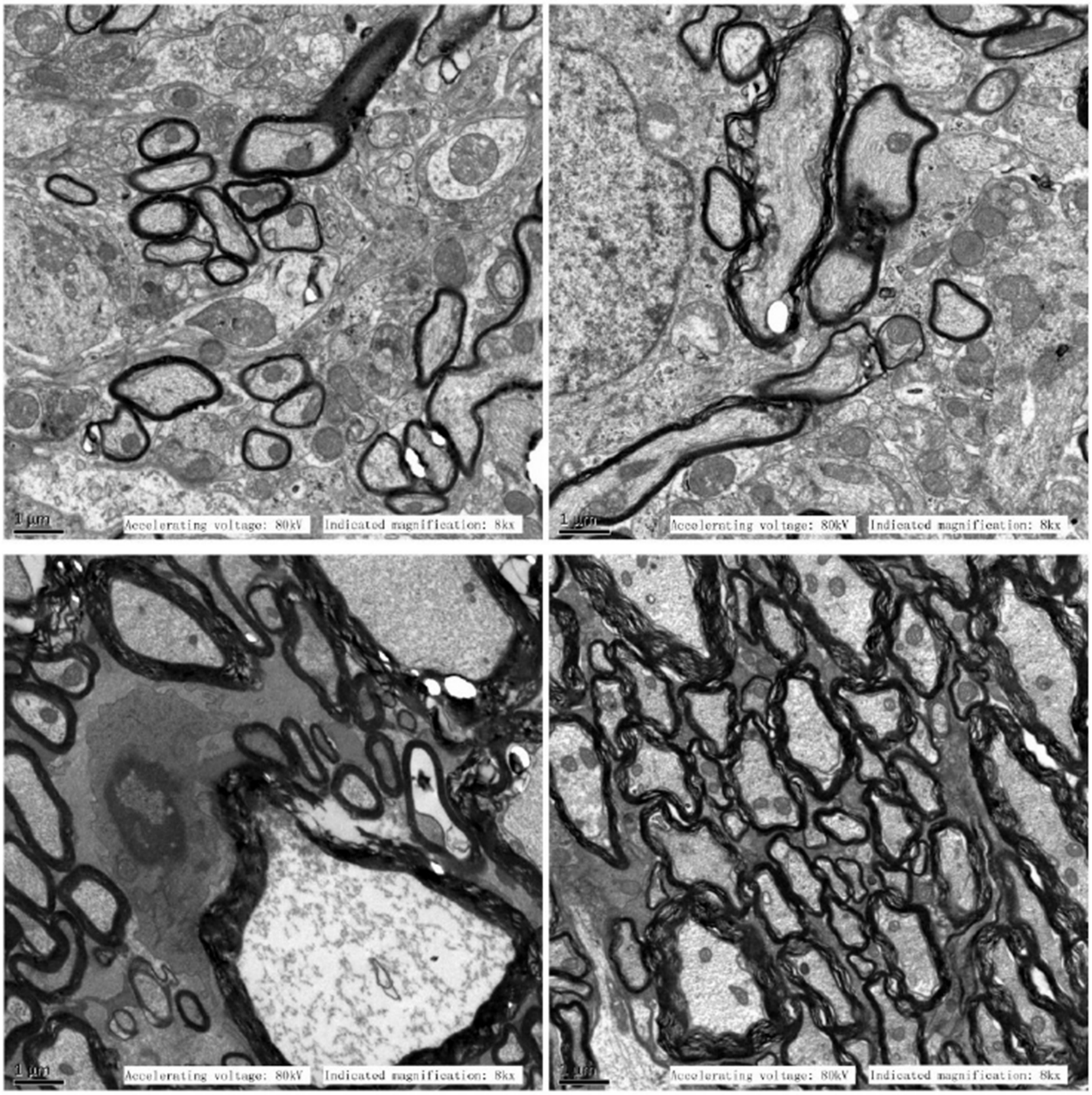

Supplementary Figure 3: Images of electron microscope for different groups. A:controlB:EAE 13dC:EAE 20dD:EAE 30d.

Supplementary Table 1: The mobility in the different experimental groups

Group	mobility	onset time	peak time
EAE	100%	12.58±1.62	18.18±1.40
SB203580	41.7%*	11.73±1.22*	15.82±0.44*
TEMPOL	75.0%*	10.81±0.50*	14.32±1.19*

*P<0.05 vs. the EAE group. n=12 per experimental group.

Supplementary Table 2: The neurofunctional deficiency in different groups

Group	premorbid (13d)	fastigium (20d)	paracmastic (30)
EAE	2.08±1.98	7.17±1.64	4.33±1.9
SB203580	0.08±0.29*	1.67±2.83*	0.83±1.59*
TEMPOL	0*	3.25±2.38*	1.17±0.94*

*P<0.05 vs. the EAE group. n=12 per experimental group.

Supplementary Table 3: Comparison of mean score of inflammation at different time points in the various experimental groups

Group	Premorbid (13d)	Fastigium (20d)	Paracmastic (30d)
EAE	1.00±0.69	2.39±0.50	0.67±0.49
SB203580	0.11±0.32*	0.44±0.62*#	0.17±0.38*
TEMPOL	0.33±0.49*	1.06±0.80*	0.28±0.46*

*P<0.05 compared with EAE group; #P<0.05 compared with TEMPOL group. n = 6 per experimental group.

Supplementary Table 4: Comparison of mean score of demyelination at different time points in the various experimental groups

Group	Premorbid (13d)	Fastigium (20d)	Paracmastic (30)
EAE	0.83±0.62	2.22±0.43	2.78±0.43
SB	0.06±0.2▲	0.56±0.62▲	0.83±0.92▲
TEMPOL	0.22±0.43▲	1.06±0.80▲	0.89±1.08▲

▲compared with EAE group, $P<0.05$ (n=6 per experimental group).

Supplementary Table 5: Comparison of mean counts of IL-17 immunopoisitive cells in spinal cords at different time points in the EAE group

Group	Mean score of IHC
Premorbid (13d)	11.33±1.97
Fastigium (20d)	26.17±2.64*
Paracmastic (30d)	12.17±0.98

*compared with other groups, P<0.05; n = 6 per group.

Supplementary Table 6: Comparison of mean counts of IL-17 immunopoisitive cells in spinal cords in different experimental groups on day 20

Group	Mean score of IHC
EAE	26.17±2.64
SB203580	6.83±1.17*
TEMPOL	7.67±1.21*

*compared with other groups, $P<0.05$; n = 6 per group.

Supplementary Table 7: Concentration of MDA in the spines at different time points

Group	MDA
Control	18.40 ± 0.96
Premorbid (13d)	34.97 ±1.84 *
Fastigium (20d)	40.30±1.61 *#
Paracmastic (30d)	33.59±2.00*

*compared with control group, P<0.05 (n = 3 per group); #compared with EAE 13d and 30d, P<0.05.
